# Olive Leaves Extract from Algerian Oleaster (*Olea europaea* var. *sylvestris*) on Microbiological Safety and Shelf-life Stability of Raw *Halal* Minced Beef during Display

**DOI:** 10.3390/foods8010010

**Published:** 2018-12-26

**Authors:** Djamel Djenane, Diego Gómez, Javier Yangüela, Pedro Roncalés, Agustín Ariño

**Affiliations:** 1 Laboratoire de Qualité et Sécurité des Aliments (LQSA), Faculté des Sciences Biologiques et des Sciences Agronomiques, Université Mouloud MAMMERI, B.P. 17, 15000 Tizi-Ouzou, Algeria; 2 Instituto Agroalimentario de Aragón—IA2 (Universidad de Zaragoza—CITA), Facultad de Veterinaria, C/Miguel Servet 177, 50013 Zaragoza, Spain; dgl.15@hotmail.com (D.G.); jyangu@unizar.es (J.Y.); roncales@unizar.es (P.R.); aarino@unizar.es (A.A.)

**Keywords:** wild olive tree, by-products, leaf extracts, High Performance Liquid Chromatography/Diode Array Detector (HPLC/DAD), *Halal* minced beef, retail/display, safety, *Escherichia coli* O157:H7, *Salmonella enterica* ser. Enteritidis, shelf-life

## Abstract

Oleaster (wild olive tree) by-products represent a renewable and low-cost source of biopolyphenols. Leaf extracts (sylv.OLE) of Algerian oleaster, locally called *a’hachad* (*Olea europaea* subsp. *europaea* var. *sylvestris*), were applied at 1 and 5% (v/w) to raw *Halal* minced beef (HMB) in order to test its safety and shelf-life prolongation during retail/display. The total phenolic compound content in the extract was 198.7 ± 3.6 mg gallic acid equivalent. Ten compounds were identified in the sylv.OLE by High Performance Liquid Chromatography/Diode Array Detector (HPLC/DAD), of which oleuropein was the most abundant (43.25%). Samples treated with 5% sylv.OLE had significantly higher antimicrobial and antioxidant effects than those treated with 1% extract (*p* < 0.05). The addition of sylv.OLE reduced psychrotrophic counts as well as the level of pathogens (*Salmonella enterica* ser. Enteritidis and Shiga toxin-producing *Escherichia coli* O157:H7). A thiobarbituric acid reactive substance (TBARS) value of 2.42 ± 0.11 was reached throughout six days of retail/display in control samples, while the addition of 5% sylv.OLE reduced TBARS value by 58% (*p* < 0.05). The presence of sylv.OLE at the tested concentrations did not negatively influence the overall acceptability and bitterness of HMB.

## 1. Introduction

In Spain, the real importance of *Halal* markets is high, and the global demand for meat slaughtered for Muslim consumption is expected to grow even faster. *Halal* minced beef (HMB) is widely consumed in many forms because of its popularity among immigrants, and for its attractive price. However, if conditions are favorable for microbial growth, minced beef can present a health risk to consumers. Pathogenic shiga toxin-producing *Escherichia coli* (STEC) O157:H7 and *Salmonella enterica* ser. Enteritidis have been reported as the major public health concerns, and have been the cause of a series of outbreaks in Europe linked to eating undercooked minced beef sold from fast-food restaurant chains and sometimes directly sold from local butchers [1]. 

The olive tree (*Olea europaea*, Oleaceae) is one of the plants mentioned in the Holy Quran (By the Fig and the Olive ^(verse1)^, by Mount Sinai ^(verse2)^, and by this City of Security ^(verse3)^: Coran, Surah 95/At’tin), and provided huge economic assets. The excellent properties of olive biophenols are a consequence of their function in the olive tree. Phenolic compounds are often involved in plant-based defense mechanisms against predation by insects, herbivores, and against multiple microbial infections. They are also responsible for the sensory properties of plants such as color, taste, and sometimes smell. In herbal medicine, the effect of olive leaves is attributed in whole, or in part, to their phenolic compounds [2]. These substances have biological activities that make them beneficial to human health. Many studies indicate that polyphenols can reduce the risk of a number of pathologies, especially those related to aging and oxidative damage (cancer, cardiovascular, or neurodegenerative diseases) [3,4]. These scientific findings have already led to approved health claims. These substances can also be used to improve the safety, shelf-life, and to develop functional foods [5]. Conditions in the Algerian region favor the growth of wild olive trees, which have provided Algerian people with dietary and economic benefits since ancient years. The “*Kabylie*” region is the most productive Algerian region of oleaster olive oil. Once the oil is extracted, the waste is often stored and released into the wild. However, by-products of oleaster leaves are a good source of added value biophenols that can be used in the food industry [6,7,8]. In this context, the recovery of waste from the olive industry seems to be an interesting market since it would respond to a current environmental problem [9,10]. Wild olive trees (oleaster) are distinguished from cultivated ones by a long juvenile period and a greater capacity for survival in difficult environments. Oleaster contains small fruits characterized by a slower mesocarp/stone ratio, it gives much lower yields in oils compared to cultivated olive trees. Several works on the biological activity of olive leaves have been carried out [5,6,11]. These effects are mainly attributed to the presence of main polyphenols in olive leaves such as secoiridoids (ligstroside, oleuropein), verbascoside, phenolic alcohols (hydroxytyrosol, tyrosol), and flavonoids (luteolin-7-glucoside, luteolin, rutin, and apigenin-7-glucoside). The majority of studies to date regarding the biological effects of olive leaves in the food field are usually in vitro studies [12,13]. 

In order to promote local products, Algerian wild olive leave extracts were used as source of natural biophenolic compounds for the enrichment of HMB commonly used for various practical meals by the Muslim community, particularly those living in Spain. Supplementation was performed at two different levels (1 and 5%, v/w) on microbiological safety and shelf-life during retail/display.

## 2. Materials and Methods

### 2.1. Olive Leaves Material

Fresh leaves from wild olive trees (locally called: *a’hachad*: *Olea europaea* subsp. *europaea* var. *sylvestris*) were collected after fruit harvesting during the period of February 2010–2012 in Ghoumrassa (Bordj-Ménaïel city: Kabylie, Algeria), from trees that have never been treated with phytosanitary products. The collected leaves were not the residues resulting from olive tree pruning, but the leaves obtained after the washing and cleaning of the olives at their entrance into the oil mill. For this, samples of leaves were collected from different parts of the trees in the same locality. The quantity of these oleaster by-products was estimated in Algeria at ≈ 5% of the weight of olives. Harvested leaves were immediately transferred to the laboratory, rinsed thoroughly with sterile distilled water to remove dust and contaminating material, and air dried at room temperature (~28 °C) during two months. After drying, the dried leaves were immediately vacuum packed, and stored in dark at 1 °C ± 0.5 °C until their use in the extraction processes during the experimental period mentioned above.

### 2.2. Preparation of Powder and Olive Leaves Extract

The packaged dried olive leaves were ground into powder (size of ~0.1 mm). The extraction was carried out by maceration of the olive leaf powder in the methanol/water (80:20, v/v), and the mixture was kept under agitation for 24 h. Insoluble material was removed by centrifugation at 15,000× *g* for 40 min. Subsequently, the clear supernatant was obtained after evaporation of the solvent by a rotary evaporator under vacuum at 40 °C. Then the supernatant was filtered (0.45 μm) to obtain the olive leaves extract, which was stored in light protected glass vials at −20 °C until further High Performance Liquid Chromatography (HPLC) analysis and use. 

### 2.3. Estimation of the Total Phenolic Compounds in the Olive Leaves Extract

Total phenolic compounds (TPC) in the sylv.OLE (extract from leaves of Algerian oleaster) were determined according to the Folin–Ciocalteu method [14] with some modifications. Two and a half milliliter (2.5 mL) portions of the Folin-Ciocalteu reagent (Sigma–Aldrich, Barcelona, Spain) were added to aliquots of 0.5 mL of extract, and incubated in the dark for 10 min (reaction). Then, 2 mL of a sodium carbonate solution (75 g/L) was added to the mixture, and the reaction was kept in the dark for 45 min. The assay tubes were vortexed and incubated at 45 °C for 10 min, and then cooled. For the blank assay, 0.5 mL of distilled water was used. Mixture absorbance was then read at 760 nm using a Jasco V-360 spectrophotometer (Jasco, Tokyo, Japan). Gallic acid was used as phenolic compound standard for the calibration curve, and TPC in sylv.OLE were expressed as milligrams of Gallic acid equivalents per gram of sample dry weight (mg GAE/g). Data were presented as the average of triplicate analyses.

### 2.4. Analysis of Phenolic Compounds in sylv.OLE

The constituents of the sylv.OLE extract were performed with an analytical High Performance Liquid Chromatography unit (Agilent Technologies 1200 series, Santa Clara, CA, USA), equipped with a diode array detector (HPLC/DAD). The stationary phase was an Atlantis® Waters dC18 column (5 μm, 4.6 × 250 mm). The elution was carried out in gradient mode using a binary solvent mixture composed of water, acidified with 5% formic acid (solvent A), and 95% methanol (solvent B) with a total analysis time of 50 minutes. The column was re-equilibrated for 4 min between each analysis. The flow rate of the mobile phase was 1 mL/min, and the injection volume of the extract sample was 10 μL. The UV absorption spectra of the standards as well as the samples were recorded in the range of 230–400 nm. Peaks of the phenolic compound were identified by comparing their retention times with those of the standards, and checking their characteristic spectra. Quantification of phenolic compounds was done by calibration curves relative to external standards, developed by injecting different amounts of a known standard compound in the HPLC column.

### 2.5. In Vitro Antioxidant Activity

The radical-scavenging activity (RSA) was measured using the stable radical 2,2-diphenyl-1-picrylhydrazyl (DPPH•) in methanolic solution according to spectrophotometric methods described by Taoudiat et al. [15]. The percentage of DPPH• radical scavenging activity (RSA) was calculated using the following Equation:

RSA (%) = [(Abs_(DPPH)_ – Abs_(sylv.OLE)_)/Abs_(DPPH)_] × 100 (1)

- Abs _(DPPH)_ = absorbance value at 517 nm of the methanolic solution of DPPH.
- Abs _(sylv.OLE)_ = absorbance value at 517 nm for the sylv.OLE.

The RSA was also expressed as the IC_50_ value (μg/mL); the concentration required to cause 50% of DPPH inhibition. To standardize DPPH results, the antioxidant activity index (AAI), proposed by Scherer and Godoy [16], and was calculated as follows:

AAI = DPPH concentration in reaction mixture (μg/mL)/IC_50_ (μg/mL) (2)

- AAI < 0.5 (poor antioxidant activity).
- 0.5 < AAI < 1.0 (moderate antioxidant activity).
- 1.0 < AAI < 2.0 (strong antioxidant activity).
- AAI > 2.0 (very strong activity).

### 2.6. Application in Halal Minced Beef

**Figure 1:**
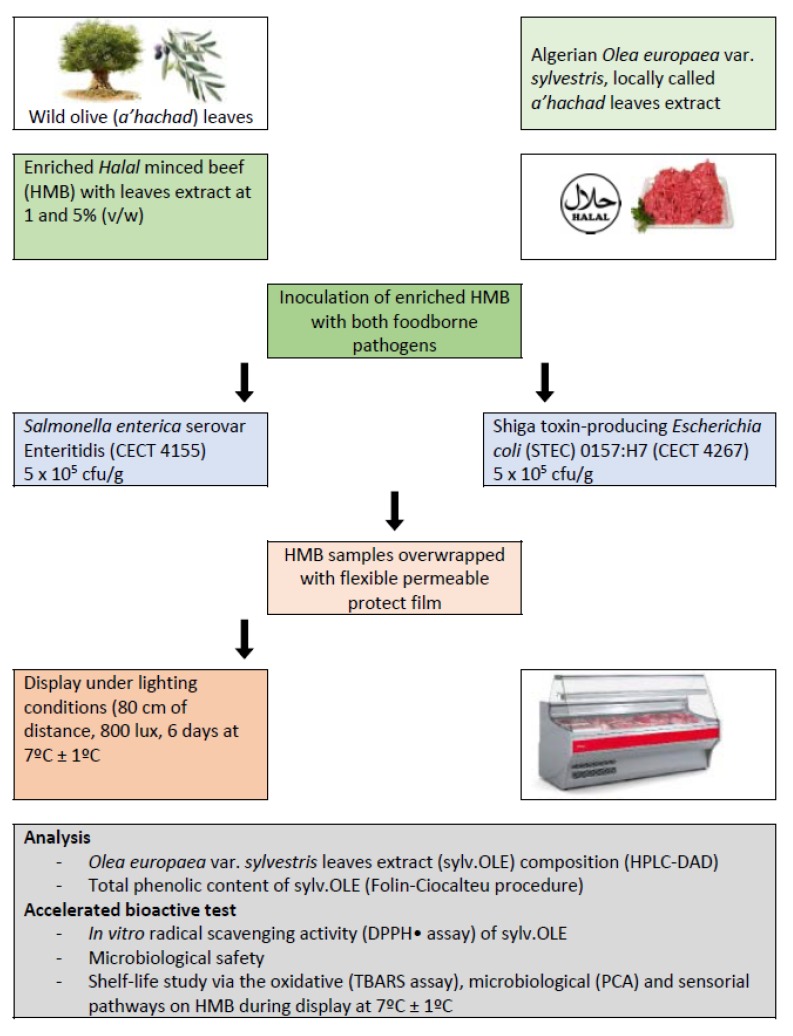
Experimental design: Sylv.OLE (extract from leaves of Algerian oleaster) application in *Halal* minced beef. HMB: Halal minced beef; STEC: shiga toxin-producing *Escherichia coli*; CECT: Colección Española de Cultivos Tipo (Spanish Type Culture Collection); HPLC: High Performance Liquid Chromatography; DAD: Diode Array Detector; DPPH•: stable radical 2,2-diphenyl-1-picrylhydrazyl; TBARS: thiobarbituric acid reactive substances; PCA: Plate Count Agar.

The experimental design is shown in Figure 1. Briefly, the extract was obtained from olive leaves of Algerian oleaster (sylv.OLE), and lots of Halal minced beef (HMB) samples were supplemented with 0 (control), 1, and 5% of sylv.OLE. Two lots of HMB were inoculated with pathogenic bacteria (Salmonella and *E. coli*) and maintained at 7 °C during display for 6 days. The sylv.OLE was chemically characterized and HMB samples were analyzed for microbial, oxidative, and sensory characteristics.

#### 2.6.1. Microorganisms and Standardization of Inoculum

The foodborne pathogens used in this work were; *S. enterica* ser. Enteritidis (CECT 4155) and Shiga toxin-producing *Escherichia coli* O157:H7 (CECT 4267), which were acquired from Spanish Type Culture Collection (Colección Española de Cultivos Tipo: CECT). To standardize the number of cells, a tube with sterile brain heart infusion (BHI) broth (Oxoid Ltd. Ref: CM0225) was inoculated from each strain by adding a bead from cryovials (Deltalab, Barcelona, Spain: Ref. 409113/6) stock stored at −80 °C (Department of Food Safety, Veterinary Faculty of Zaragoza, Spain). After incubating of the tubes at 37 °C for 12–16 h, their concentrations were measured by spectrophotometry (Spectronic 20, Bausch & Lomb, Rochester, NY, USA) after carrying the reading at 59% transmittance by culture combination and sterile BHI. For each tube with the adjusted transmittance, counts were made on agar plates for plate count (PCA) by seeding 1 mL of the dilutions −5, −6, and −7 to verify the veracity of the spectrophotometer readings. The final bacterial loads were 1.8 × 10^8^ CFU/mL for *S. enterica* ser. Enteritidis, and 3.5 × 10 ^8^ CFU/mL for *Escherichia coli* O157:H7.

#### 2.6.2. *Halal* Minced Beef and Treatments

Raw *Halal* minced beef samples were purchased on 3 occasions, during the period of December 2010 through September 2012 from different *Halal* butchers’ at Zaragoza city, Spain. Meat samples were aseptically transported under cool conditions (1 ± 1 °C) to the laboratory of Food safety, Veterinary Faculty of Zaragoza (Spain) within 30 min. The total quantity of minced meat purchased was strictly maintained at 1 ± 1 °C and all subsequent preparations were held in a cold room at 2 ± 1 °C. Samples were divided into various small lots, and were subjected to different treatments. Individual samples of about 100 g weight were aseptically processed, placed into sterile stomacher bags, and contaminated separately with each pathogen (final concentration: ~5.0 × 10^5^ CFU/g). In order to ensure proper distribution of the microorganisms, the inoculated samples were homogenized in the stomacher (400 circulator, Seward) for 2 min. Following homogenization, sylv.OLE was added to the inoculated samples. The following treatments of inoculated minced meat samples were used: Untreated (control), and addition of sylv.OLE (1 and 5%, v/w). To ensure uniform distribution of the added compounds, treated meat samples were further homogenized in the stomacher as previously described. The stomacher bags with samples from all treatments were sealed and placed into polystyrene trays of size 15.5 × 21.5 × 2.5 cm. All trays were stored in refrigerated cabinets under illumination at ~7 ± 1 °C for 6 days, simulating proper refrigeration retail/display. The initial microbiological quality was also determined in purchased HMB prior to laboratory studies. Three samples from each group were taken at each selected time (0, 2, 4, and 6 days) for subsequent analysis.

#### 2.6.3. Microbiological Analysis

For the enumeration of *Salmonella enterica* ser. Enteritidis, 25 g of HMB were added in duplicate to 100 mL of buffered peptone water (BPW; Oxoid Ltd. (Hampshire, UK) Ref: CM1049; dilution 1:5) in a sterile plastic stomacher filter bag (Seward). The mixture was homogenized in the stomacher for 1 min, and 0.33 mL were plated in triplicate onto ChromID™ *Salmonella* agar (BioMérieux SA, Marcy l’Etoile, France, Ref: 43621), which was incubated at 37 °C for 18–24 h. 

For enumeration of *E*. *coli* O157:H7 by direct plating, 25 g of HMB were weighed in duplicate in a volume of 100 mL of modified tryptone soya Broth (Oxoid Ltd. Ref: CM0989) + novobiocin (Oxoid Ltd. Ref: SR0181). The mixture was homogenized in the stomacher during 1 min, and 0.33 mL were plated in triplicate onto ChromID™ O157:H7 agar (BioMérieux SA, Ref: 42605), which was incubated at 37 °C for 18–24 h.

For the enumeration of psychrotrophic bacteria, 25 g from each individual sample of HMB were taken and diluted in 225 mL 0.1% peptone water (PW). The mixture was homogenized in a stomacher for 1 min. Serial 10-fold dilutions were prepared by diluting 1 mL in 9 mL of 0.1% PW. Three plates were prepared from each dilution by pouring 1 mL in fluid agar. Counts of aerobic psychrotrophic bacteria were determined in plate count agar (PCA; Merck, Darmstadt, Germany) and incubated at 7 °C for 10 days. Counts were expressed as log_10_ of colony forming units/g (CFU/g). The logs of mean values for the counts from triplicate plates were recorded.

#### 2.6.4. Malonaldehyde Compounds as Lipid Oxidation Biomarkers in HMB

During the display period, the degree of lipid oxidation was determined using 2-thiobarbituric acid (TBA) to measure secondary oxidation products, described by Djenane et al. [17]. The results were expressed as mg malonaldehyde (MDA)/kg of product and calculated using a standard curve prepared with 1,1,3,3-tetramethoxypropane (Sigma Aldrich Corporation, St. Louis, MO, USA).

#### 2.6.5. Sensory Evaluation of *Halal* Minced Beef

Only sylv.OLE control samples were subjected to evaluation of their sensory properties, using the method described by the American Meat Science Association guidelines [18]. Eight (08) panelists were selected from laboratory staff (Laboratory of Food Science, University of Zaragoza, Spain). Panelists evaluated the fresh meat “*off-odor*” referred to the intensity of odors associated to both oxidation and microbiological product spoilage, using a numerical scale of 1–5 (with 1 = none and 5 = extreme), as well as the bitterness by using a numerical scale of 1–5 (with 1 = no bitterness and 5 = very bitter). Finally, panelists evaluated the “*overall acceptability*” of each sample taking into account any “*off-flavor*” and undesirable taste, using a numerical scale of 1–5 (with 1 = very acceptable and 5 = unacceptable). For bitterness and overall acceptability evaluation, HMB were cooked for 30 min, and served warm to panelists. Each sample was presented with a three-digit code randomly chosen. A score value higher than 3 of any attribute, denoted that minced beef was not acceptable by panelists probably due to end of shelf-life. Three samples from each group were taken at each selected time (0, 2, 4, and 6 days) for subsequent sensorial analysis.

#### 2.6.6. Statistical Analysis

Results were presented as the means of three independent experiments conducted at least in triplicate ± standard deviation for each experimental design. The data were analyzed by t-student’s test and ANOVA to check the effects of treatment and time of storage (0, 2, 4, and 6 days) using the Statistical Package for the Social Sciences software (SPSS version 21, IBM Corporation, Armonk, NY, USA). The effect of the sylv.OLE treatment (1–5%) on HMB quality and microbiological safety was evaluated during display period with a significance level of *p* < 0.05.

## 3. Results and Discussion

### 3.1. Total Phenolic Contents

In 1 g of dry weight of sylv.OLE, 198.7 ± 3.6 mg gallic acid equivalent was detected by the Folin–Ciocalteu assay (Table 1). These results suggest that the higher levels of antioxidant activity were due to the presence of phenolic components. Phenolic compounds are known to be good natural antioxidants and are widely investigated in many vegetables and medicinal plants [19]. The total phenolic content in our sylv.OLE was in agreement with Altemimi et al. [20], who found values between 147.78 ± 0.69 and 190.44 ± 0.50 mg/g in Iraqi olive leaves. According to scientific literature, the content of phenolic compounds in olive leaves varies between 2.8–44.3 mg/g of dry matter [21,22,23], but it can even exceed 250.2 mg/g [24]. The variation in the concentration of phenolic compounds in olive leaves indicated in the literature depends on climate and agronomic conditions, olive cultivar, soil composition, the time of collection of leaf samples, and the age of tree. In addition to these variability factors, there are the effects of storage conditions prior to extraction, method of preparation of olive leaves, and the processing techniques. For example, Boscaiu et al. [25] found that the level of phenolic compounds accumulated in plants and stress (biotic and abiotic) were positively correlated, suggesting a role of these secondary metabolites in the defense mechanisms against this phenomena.

### 3.2. HPLC Analysis

A chromatographic analysis, by High Performance Liquid Chromatography/Diode Array Detector (HPLC-DAD), was carried out in order to determine the phenolic composition in the sylv.OLE. Ten compounds were identified in this work (Table 2). The quantitative results of the 10 compounds identified showed that the most abundant compound was oleuropein (43.25%), as found by various authors [17,26,27,28,29,30,31,32]. In addition to oleuropein, other phenolic compounds were identified: verbascoside (13.12%), apigenin-7-glucoside (9.82%), luteolin-7-glucoside (1.34%), hydroxytyrosol (7.32%), rutin (0.76%), vanillic acid (0.65%), luteolin (0.89%), tyrosol (3.15%), and caffeic acid (0.79%). The quantitative analysis of our sylv.OLE showed notable differences compared to that obtained from non-sylvester olive leaf extract (OLE) in different regions of the world. Lee-Huang et al. [28] analyzed the OLE and found that this extract contains; oleuropein (12.8%), verbascoside (0.38%), luteolin-7-glucoside (0.68%), rutin (0.34%), apigenin-7-glucoside (0.18%), and luteolin (0.41%). Botsoglou et al. [29] and Hayes et al. [12] quantified various polyphenols in OLE and also reported that oleuropein was the largest fraction present (43.58%), while other polyphenols like hydroxytyrosol (4.56%), luteolin-7-glucoside (21.54%), verbascoside (25.12%), tyrosol (6.58%), and apigenin-7-O-glucoside (30.22%) were also isolated from the leaves. However, Pereira et al. [33] reported that oleuropein and lueolin-7-O-glucoside were the most abundant compounds in a lyophilised OLE. Altiok et al. [21] also quantified oleuropein (29%) as the major phenolic compounds present in crude OLE. Compared to olive oil and oil mill waste water, olive leaves contain higher oleuropein, which ranges from 1 to 14% [34,35]. It is well known that the phenolic compositions of olive leaves and subsequent antiradical scavenging are considerably affected by the biological cycle of olive trees, the environmental and agronomical conditions, such as climate, region, and furthermore by point of harvest, ripeness and post-harvest processing, long-term storage stability of dried olive leaves, but also by the methods of sample preparation and extraction and by the method of quantification [36,37]. 

Many studies have evaluated the therapeutic role of oleuropein and its mechanisms of action. Extensive scientific research has shown that oleuropein has antioxidant [38] and antimicrobial activities [39,40]. The concentration of oleuropein in studied sylv.OLE can reach up to 43% of dry leaves. As a result, the oleaster is considered a renewable natural source for the extraction of oleuropein. However, to our knowledge, there is little discussion in the literature concerning the possibility to add the sylv.OLE to minced beef in order to increase its shelf-life and for safety considerations. Some works have already demonstrated that biophenols encountered in olive leaves also display a synergistic behavior in their biological activities when used together, showing that the mixture of major and minor bioactive compounds is higher than that of the individual phenolics alone. 

### 3.3. In Vitro Antioxidant Activity

Radical scavenging activity of the sylv.OLE natural product and a synthetic antioxidant butylated hydroxytoluene (BHT) were assessed using the 2,2-diphenyil-picrylhydrazyl (DPPH•) assay (Table 1). The sylv.OLE extracts was more effective in scavenging DPPH (IC_50_ = 19.2 ± 1.6 μg/mL) and showed a very strong antioxidant potential with AAI = 4.2 ± 0.1. Although the antioxidant activity found in vitro was only indicative of the potential food benefits, these results remain generally important as the first step in screening the antioxidant activity of sylv.OLE. These extracts owe their antioxidative properties to their high biophenol content, as they possess ideal structural chemistry for free radical scavenging activity. The leaves of sylvester *Olea europaea* are characterized by high oleuropein content, and also to a lesser amount of hydroxytyrosol. The structure of phenolic compounds is a key determinant of their radical scavenging, and their potential antioxidative activities have been known for a long time. Given sylv.OLE richness in biophenol molecules, this latter may protect the foods from free radical damage. The DPPH radical scavenging potential of olive leaf extract was also investigated by Bouaziz et al. [41], who found an IC_50_ = 1.5 μg/mL comparable to that of pure oleuropein; IC_50_ = 1.19 μg/mL. The radical scavenging activity of phenolic compounds in sylv.OLE could be due to the presence of the hydroxyl groups in their structure such as oleuropein and hydroxytyrosol. The hydroxytyrosol from OLE has previously shown to have strong DPPH radical scavenging activity [41]. Authors claimed that this biophenol may act as a good hydrogen (H) donator by scavenging free radicals. On the other hand, a good correlation between oleuropein content and antioxidant potency of OLE has been previously reported [42]. For the comparison between total phenolic compounds and scavenging free radical activities, Altemimi et al. [20] found that the plant extracts with the highest amount of phenolic content will be more effective at scavenging free radicals. The sylv.OLE and their biophenol compounds are considered candidate constituents of particular importance. These phytochemical compounds can be studied for their possible promising results with respect to their biological potential activities in the agro-food field. Combinations of OLE biophenol compounds such as hydroxytyrosol with other antioxidants revealed additive effects [43].

A process involving the usage of oleuropein and hydrolysate OLE to produce hydroxytyrosol has been developed by Zafer and Filiz [13], and Bouaziz et al. [41]. These latter found IC_50_ = 0.58 μg/mL value for OLE hydrolysate, and concluded that this antioxidant power was due to hydroxytyrosol, which resulted from oleuropein hydrolysis. On the other hand, these results can be explained by the phenomena of hydrolysis with the corresponding increase in the antioxidant activity of the extract and probably the synergistic effect between OLE biophenols [13].

### 3.4. Microbiological Analysis

#### 3.4.1. Plate Count Agar (Psychrotrophic Microbiota)

The minced meat constitutes, by their intrinsic and extrinsic qualities, are very good substrates for good and fast development of various bacteria. Compared to whole muscle, sometimes, even if hygienic conditions and the cold chain are respected, this fragile product may suffer various alterations during storage. Therefore, minimizing product contamination and delaying or inhibiting growth of spoilage and pathogenic organisms in the product are major keys for improving fresh meat shelf-life and increasing consumer safety. Among Muslims, minced meat is almost omnipresent in everyday gastronomic customs, and especially during social and religious events (e.g., marriages, circumcisions, Ramadan, Al aqîqa, etc.). Often in this community, ground beef is widely consumed in many forms. However, if conditions are favorable for microbial growth, minced beef under various practical meals can deteriorate quickly and can present a health risk to consumers. The total psychrotrophic microbiota is a good indicator for quality control and shelf-life estimation of foodstuffs of animal origin during storage. At high load of psychrotrophic microbiota is synonymous with an imminent microbiological alteration during storage.

**Figure 2:**
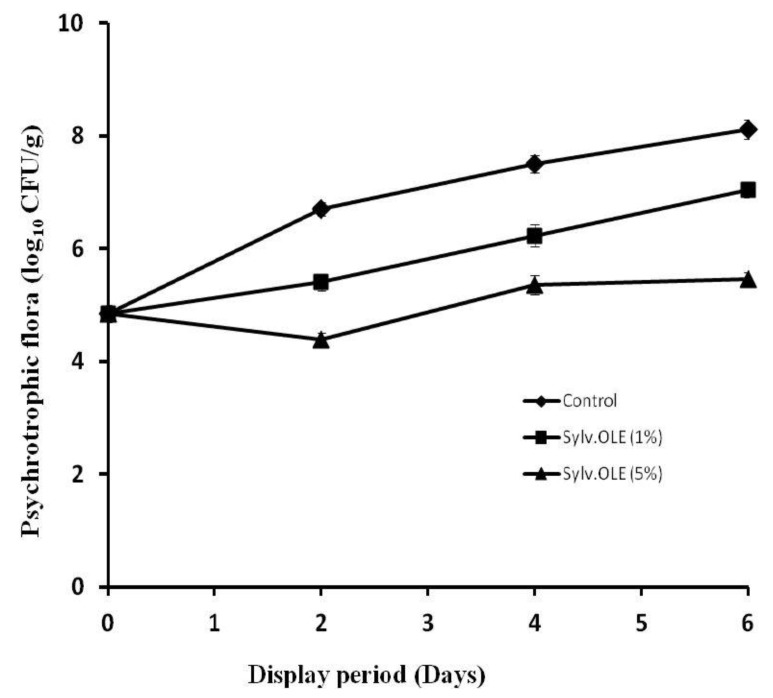
Total psychrotrophic bacteria counts (log_10_ CFU/g ± SD (standard deviation)) of HMB (Halal minced beef) treated with sylv.OLE during display.

Figure 2 shows the evolution of the psychrotrophic microbiota in treated HMB. The addition of extract to the minced meat produced a net regression of the total psychrotrophic microbiota during display period depending on the concentration used. Stronger antimicrobial activity was obtained in the presence of higher concentrations of the extract (5%), though, 1% of the extract also reduced the microbial growth compared to the control (*p* < 0.05). At higher concentrations, the reductions obtained in terms of psychrotrophic microbiota in HMB were 2.31, 2.14, and 2.66 log_10_ CFU/g at 2, 4, and 6 days of storage, respectively. From a microbiological point of view, these results have a very positive significance in terms of the microbiological stability and subsequently higher shelf-life of the product during the display period. Indeed, even at the end of the storage period, the level of the microbial load was kept very far from the critical microbiological threshold (≈7 log_10_ CFU/g = end of shelf-life). However, the control samples began to develop signs of alteration from the 2nd day of storage (6.7 log_10_ CFU/g); on the 4th day the microbial load already exceeded the critical threshold (7.5 log_10_ CFU/g). The presence of oleuropein, and to a lesser degree other minor biophenols, in sylv.OLE may partly explain the antimicrobial activity. Juven et al. [44] reported that extracts of green olives exerted an antimicrobial effect against various Gram-positive and Gram-negative bacteria. Likewise, OLE-containing oleuropein and hydroxytyrosol showed important antibacterial effects in raw shrimp by reducing the count of aerobic bacteria by at least 1 log_10_ CFU/g compared to the untreated samples [8]. Wei et al. [45] reported that these biophenols exert antimicrobial activity by destroying membrane permeability. Previously, other authors reported that dietary olive leaves were more effective than α-tocopheryl acetate supplementation (*p* < 0.05) in inhibiting microbial growth in turkey breast fillets during refrigerated storage [46].

#### 3.4.2. Pathogenic Microbiota

**Figure 3:**
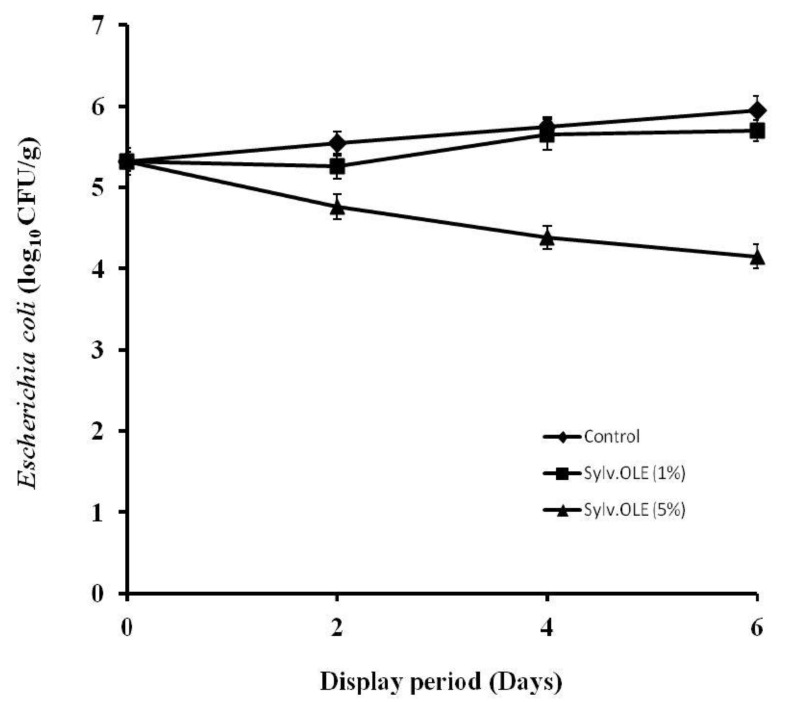
*Escherichia coli* O157:H7 growth rate in HMB during display in the presence of different sylv.OLE concentrations.

**Figure 4:**
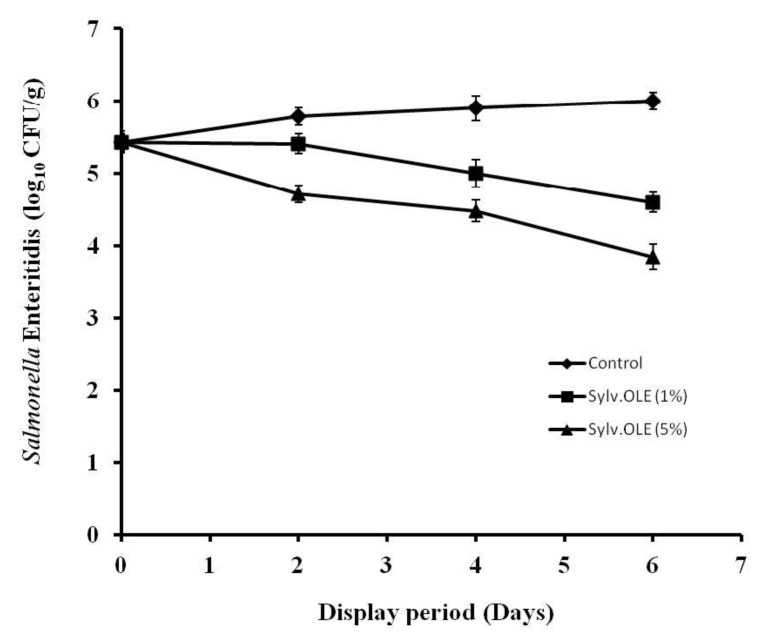
*Salmonella enterica* ser. Enteritidis growth rate in HMB during display in the presence of different sylv.OLE concentrations.

The mincing of meat in butchery *Halal* at retail/display is a particular critical process, as food borne pathogens will be physically distributed onto various surfaces due to the inadequate temperature control and poor hygiene during mincing operation. This process may lead to spread and growth of pathogens in the product. Therefore, minimizing cross-contamination and delaying growth of food borne pathogens are major keys for increasing consumer safety. There are several studies that have shown a high prevalence of foodborne pathogens in ground meat due the lack of hygiene during the different operations. On the other hand, the consumption of undercooked ground meat is recognized as a major risk factor for people infected by food-borne pathogens. Our research group has postulated the potential use of sylv.OLE for microbial safety of retailed HMB. The results showed that the type of microorganism, the concentration of sylv.OLE additives, and display periods affected the microbiological values (*p* < 0.05). The counts of pathogenic bacteria in minced meat were significantly lower in samples treated with sylv.OLE than in control samples (*p* < 0.05). As shown in Figure 3 and Figure 4, the growth of both foodborne pathogens increased over display period in control HMB samples without sylv.OLE. The bacterial load reached ~6 log_10_ CFU/g for both foodborne pathogens at the end of display. In contrast, the samples treated with 5% sylv.OLE showed lower counts for both pathogens (3.85 ± 0.05 and 4.15 ± 0.10 log10 CFU/g, respectively for *S*. Enteritidis and *E. coli*) corresponding to reductions of 1.6 and 1.2 log10 CFU/g as compared with respective initial load.

On the other hand, our results revealed that an increase in the concentration of sylv.OLE significantly decreases the bacterial load in inoculated HMB samples (*p* < 0.05). For instance, *Salmonella* reduction with 1% sylv.OLE were 0.9 and 1.40 log10 CFU/g on the 4th and 6th day of display, respectively, while 5% sylv.OLE achieved reductions of 1.38 and 2.15 log10 CFU/g at the same sampling times. Regarding *Escherichia coli*, there were no statistically significant differences between control samples and those treated with 1% sylv.OLE (*p* > 0.05). However at 5% sylv.OLE, reductions of 0.78, 1.36, and 1.80 log10 CFU/g were recorded during the 2nd, 4th, and 6th days of display, respectively. These results were consistent with those of previous works in which olive biophenols showed antimicrobial effects in the food matrix. Medina et al. [47] detected elevated death rates for *S.* Enteritidis in milk- or egg-based mayonnaise containing virgin olive oil with high biophenolic content. Hayes et al. [48] studied the antimicrobial effect of OLE on meat and reported a noticeable antimicrobial effect in products under various packaging conditions (MAP and overwrap). Radford et al. [49] conducted a study on mayonnaise containing virgin olive oil to determine the antimicrobial effect of OLE. They deduced a good anti-*Salmonella* Enteritidis activity due mainly to the polyphenol richness of this tested extract.

Antibacterial activity of OLE was also tested using agar dilutions and broth microdilution techniques, and was found to be very active against a wide range of microorganisms, with minimum inhibitory concentrations (MICs) as low as 0.31–0.78% (v/v) [50]. On the contrary, Albertos et al. [51] found antibacterial activity of OLE against *Listeria monocytogenes* (*L. monocytogenes*) in agar diffusion tests, but no effect was observed on *S. enterica* and *E. coli*. *Campylobacter* species were found to be very susceptible in vitro to OLE [50], while *Salmonella* species were the most resistant [52].

As shown in Table 1 and Table 2 sylv.OLE contains high amounts of biophenols (198.7 mg/g). Most of these phenolic compounds have been shown to possess antimicrobial activity. The bactericidal and bacteriostatic activities of oleuropein and hydroxytyrosol have been well investigated [40]. Hydroxytyrosol is the principal product of oleuropein degradation, so its amount in olive leaves increases during several treatments. Koutsoumanis et al. [39] reported that oleuropein exerted a bacteriostatic effect on *Salmonella* Enteritidis. Further research was conducted regarding the antibacterial effects of oleuropein and some studies have shown rather contradictory results regarding the antimicrobial activity of oleuropein against microorganisms that could be attributed to different methodological approaches. Medina-Martínez et al. [53] recently addressed these discrepancies and re-evaluated the activity against various pathogenic bacteria.

The effect of oleuropein concentration on *Staphylococcus aureus* (*S. aureus*) was determined by Tranter et al. [54], who found that at >0.2% (w/v) level, the growth of *S. aureus* and subsequent toxin production were sufficiently delayed, whereas at 0.6%, both metabolic parameters can be completely inhibited. Besides, Tassou and Nychas [55] observed that at reduced pH levels, the antibacterial activity of oleuropein against bacteria was increased. Most studies revealed a higher antimicrobial activity for the oleuropein aglycone compared to the oleuropein glycoside [56]. OLE showed appreciable activity against *Campylobacter jejuni* (*C. jejuni*), *Helicobacter pylori* (*H*. *pylori*), and *S. aureus*, which could indicate the role as potential bioactive compound for the regulation of gastric microbiota [49]. Recent investigations showed the antibacterial potential against *L. monocytogenes* in cold-smoked salmon packed with plastics films with OLE. Antimicrobial activity of the films was increased with increasing OLE concentration in their formulations. The active films significantly reduced the growth of this pathogen on the product during storage [51]. 

Some information is available regarding the full antimicrobial spectrum of the individual phenolics and their synergisms in combinations, as well as the interactions with food matrix ingredients. Lee and Lee [57] found that *S*. Enteritidis was fully inhibited by the combination of OLE compounds compared to the individual phenolics. Additionally, OLE compounds can be incorporated to foods in microencapsulated forms to enhance activity and preserve sensorial properties.

### 3.5. Lipid Oxidation

Lipid oxidation is the major cause of chemical alteration of animal products during processing and storage [58,59]. The oxidation reactions cause irreversible changes in the taste, flavor, color, and texture of the products, resulting in a decrease in their shelf-life. Synthetic antioxidant substances are often used on a large scale in the food industry to limit this phenomenon. Nevertheless, their use has been questioned by the potential toxicity of these substances. Then, natural antioxidants can constitute a good alternative to protect foods against lipid oxidation. Ground meat, because of its structure, has a much shorter shelf-life than other meats, even when frozen. It is more sensitive to deterioration because the unsaturated fatty acids are distributed evenly throughout the mass, which is exposed to oxygen and light. Spices and herbs are frequently used at *Halal* butchers during handling and preparation. However, chlorophyll present in these products absorb light and consequently may accelerate the rate of photo-oxidation of minced meat [60]. The antioxidant properties of the sylv.OLE and their capacity to inhibit lipid peroxidation rates in HMB were characterized in order to make them candidates as substitutes for synthetic antioxidants commonly used to increase the shelf-life of meat products during retail/display in supermarkets.

**Figure 5:**
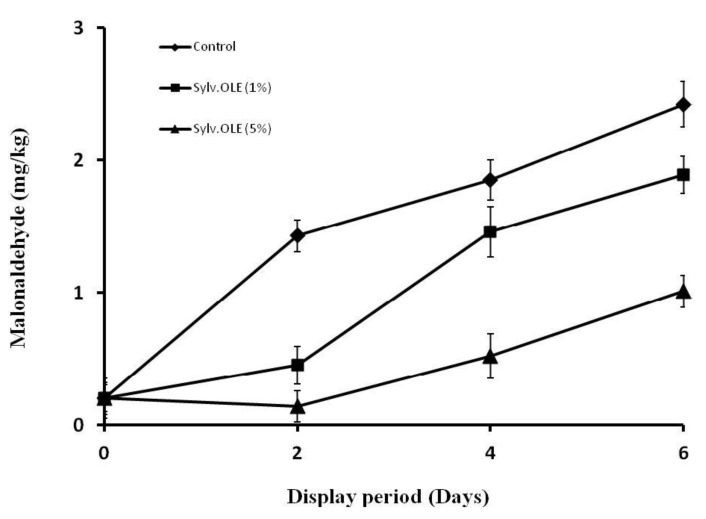
TBARS (thiobarbituric acid reactive substances) values (mg MDA/kg) during display of HMB containing different sylv.OLE concentrations.

The thiobarbituric acid reactive substances (TBARS) value of untreated HMB sample without any added sylv.OLE reached a maximum value of 2.42 mg malondialdehyde (MDA)/kg after six days of display (Figure 5). On the other hand, our results revealed that an increase in the concentration of sylv.OLE significantly decreases the TBARS value of HMB samples (*p* < 0.05). When measuring TBARS value, lipid oxidation biomarker (malondialdehyde) reacts with thiobarbituric acid. Thus, the rate of reaction increases during meat oxidation. Direct addition of sylv.OLE in HMB had a positive effect on the oxidative stability of product during display times as compared to control (untreated) samples. These results were in agreement with those of previous studies, in which fresh olive leaves or their extracts showed antioxidant effects in the food matrices, such as meat [29,46], in bovine and porcine muscle model systems [48], pork patties during frozen storage [61], and in edible oils [41,62].

Our results showed that both the display period and the concentration of additive affected the TBARS values (*p* < 0.05). The TBARS values of all the tested samples (control and treatment) increased constantly with the time of storage. The increase was the most pronounced for the control sample, whose TBARS values rose from 0.2 mg MDA on day 0, to 2.42 mg at the end of display (6th day). The TBARS value for the treatment with sylv.OLE 1% at the end of storage was 1.89 mg MDA/kg, and was significantly lower (*p* < 0.05) than the control. In samples treated with sylv.OLE 5%, the MDA value at day 6 was 1.01 mg MDA/kg, indicating a significant reduction by 58% as compared to the control.

It was observed that TBARS values in pre-cooked beef and pork can be reduced by 83% and 66%, respectively, using waste waters of olive oil pomace. A strong positive synergism between biophenols present in the extract was detected [63]. Hayes et al. [48] studied, on meat, the antioxidant effect of OLE. In comparison to control, the OLE reduced lipid oxidation by 76–84% under MAP and by 53–78% under aerobic conditions. Recently, the use of olive leaves for animal feed strategies had a positive effect on lipid oxidation and on the sensory attributes of meats during refrigerated storage [29,46]. 

### 3.6. Sensory Evaluation

The displayed, untreated HMB shows an increase in lipid oxidation products and consequently, the appearance of rancid odor after only two days of display (Table 3). The results of this study showed a good correlation between TBARS values and sensory analysis. The statistical study of sensory analysis indicated that there were no significant differences between the two addition levels of sylv.OLE (1% and 5%) during the first four days of display (*p* > 0.05). However, treated samples were significantly different (*p* < 0.05) from the control. The control samples with the highest levels of TBARS and PCA counts were ranked the most rancid by the sensory panel. In addition, the evaluation of the acceptability by the panelists allowed an estimation of the shelf-life of HMB (Table 4). A recent study on the odor perception of beef steaks, according to their state of freshness, related to the off-odors with the evolution of the concentrations and the nature of the lipid oxidation compounds such as malonaldehyde [17]. The study showed that during refrigerated storage under light, beef steaks gradually lost their fresh smell characteristics to acquire rancid notes. The interest of the olive leaves has been mainly correlated with its content in oleuropein. However, this compound may be responsible for the bitter taste of various products [64]. For this, an investigation of the sensory impact of sylv.OLE on minced meat was carried out. The sensory evaluation of the treated HMB showed a decrease in bitterness during display, as samples containing 1% or 5% of sylv.OLE extract (85.94 mg/g oleuropein) were accepted in terms of less bitterness. Moreover, in the evaluation of overall acceptability, panelists expressed a clear acceptability towards samples treated with 5% sylv.OLE. Regarding the stability of biophenol compounds in food applications, Zoidou et al. [65] found that the incorporation of olive biophenol into crude and fermented animal products was not affected during heat treatment. On the other hand, the trend to use plant extracts in foods as natural preservatives, may also influence health of consumers [66]. 

## 4. Conclusions

Oleaster leaves, an abundant and low cost raw material, are by-products of the traditional Algerian olive oil extraction process with high potential added value. Large amounts of oleaster leaves are collected during the admission to the oil mill at the first processing stage. The total phenolic compounds content in oleaster leaves extract was 198.7 mg gallic acid equivalents (GAE), and ten compounds were identified of which oleuropein (43%), verbascoside (13%), apigenin-7-glucoside (10%), and hydroxytyrosol (7%) were the most abundant.

Our results showed for the first time that the leaves from oleaster are a great source of biophenols, which could be used as natural bioactive compounds in food applications. Halal minced beef (HMB), commonly used for various practical meals by Muslim communities living in Spain, was supplemented with oleaster leaves extract (sylv.OLE) at two levels (1% and 5%, v/w), and tested for improved microbiological safety and shelf-life during retail display at 7 °C for six days. Our results of higher levels of antioxidant and antimicrobial activities in supplemented HMB, suggest that the presence of bioactive compounds in oleaster leaves extract was very effective. 

Additionally, it has been shown that the presence of this extract at the indicated addition levels did not have a negative influence either on the overall acceptability and bitter taste of treated HMB. However, there is a need for further studies regarding the synergistic behavior with other antimicrobial compounds, such as nisin, and for the application by active packaging techniques in order to produce optimized effects.

## Figures and Tables

**Table 1:** Total phenol content and free-radical scavenging activity of leaves extract from Algerian oleaster (*O. europaea* var. *sylvestris*).

Method	Parameters	Sylv.OLE	Standard BHT
Free-radical scavenging activity ^1^	- IC_50_ (μg/mL) ^2^ - AAI ^3^ - Antioxidant activity	19.2 ± 1.6 4.2 ± 0.1 Very strong	11.4 ± 0.5 7.1 ± 0.2 Very strong
Folin-Ciocalteu	- Phenol content (mg/g)	198.7 ± 3.6	

^1^ DPPH• scavenging method (mean ± standard deviation (SD) of three determinations). ^2^ IC_50_ value: Concentration required causing 50% of DPPH inhibition. A lower IC_50_ value indicates greater antioxidant activity. ^3^ AAI (Antioxidant Activity Index): AAI = DPPH concentration in reaction mixture (μg/mL)/IC_50_ (μg/mL); DPPH concentration in reaction mixture = 80 μg /mL; IC_50_ values are defined as the concentration of test material, which is able to decrease the initial concentration of DPPH to half of its initial value; AAI < 0.5 (Poor); 0.5 < AAI < 1.0 (moderate); 1.0 < AAI < 2.0 (strong), and AAI > 2.0 (very strong). BHT: butylated hydroxytoluene; DPPH•: Stable radical 2,2-diphenyl-1-picrylhydrazyl; Sylv.OLE: extract from leaves of Algerian oleaster.

**Table 2:** Phenolics composition (%) in Algerian sylv.OLE (extract from leaves of Algerian oleaster).

Phenol Compounds	Composition (%)
**1.** Oleuropein	43.25 ±1.26
**2.** Verbascoside	13.12 ± 0.96
**3.** Apigenin-7-glucoside	9.82 ± 1.06
**4.** Hydroxytyrosol	7.32 ± 0.58
**5.** Tyrosol	3.15 ± 0.09
**6.** Luteolin-7-glucoside	1.34 ± 0.08
**7.** Luteolin	0.89 ± 0.03
**8.** Caffeic acid	0.79 ± 0.04
**9.** Rutin	0.76 ± 0.01
**10.** Vanillic acid	0.65 ± 0.01

Mean ± SD (standard deviation) of three determinations by the HPLC-DAD method.

**Table 3:** Sensory scores* of treated and untreated *Halal* minced beef (HMB) during period of display.

Parameters	Treatments	Days of Display			
		0	2	4	6
*Bitterness*	Control HMB (1%) HMB (5%)	1.00(0.00) 1.88(0.35) 2.63(0.52)	1.00(0.00) 1.75(0.46) 2.63(0.52)	1.00(0.00) 1.63(0.52) 2.38(0.52)	1.00(0.00) 1.75(0.46) 2.00(0.53)
*Off*-*odor*	Control HMB (1%) HMB (5%)	1.00(0.00) 1.00(0.00) 1.00(0.00)	2.63(0.52) 2.38(0.52) 2.13(0.35)	3.25(0.46) 2.63(0.52) 2.25(0.46)	4.00(0.53) 3.13(0.35) 2.25(0.46)
*Overall acceptability*	Control HMB (1%) HMB (5%)	1.00(0.00) 1.00(0.00) 1.25(0.46)	2.63 (0.52) 2.00 (0.53) 2.00 (0.53)	3.38(0.52) 2.63(0.52) 2.38(0.52)	4.25(0.46) 3.63(0.52) 2.50(0.53)

Values represent the scale of studied parameters (1–5) and the mean and standard deviation (in parenthesis) of eight observations. *A score value ≥3 of any attribute, denoted that minced beef was not acceptable by panelists due to the end of the shelf-life. HMB (1%): *Halal* minced beef treated with sylv.OLE at 1%. HMB (5%): *Halal* minced beef treated with sylv.OLE at 5%.

**Table 4:** Shelf-life (days) determination.

Sample Type	Shelf-Life Period (days) at 7 °C
Control	2
Sylv.OLE (1%)	4
Sylv.OLE (5%)	>6

^∗^ Shelf-life = the period during which the product is in satisfactory quality state. It is determined based on sensory analysis (attribute scoring: A score ≥3 in any of the parameters denoted that meat was unacceptable for sale or consumption), chemical (thiobarbituric acid reactive substances (TBARS) value: Limit 1.5 mg MDA/kg) and microbiological (Psychrotrophic aerobic count: Limit 7 log_10_ CFU/g) criterion.
